# Pre-operative cellular dissociation grading in biopsies is highly predictive of post-operative tumour stage and patient outcome in head and neck squamous cell carcinoma

**DOI:** 10.1038/s41416-019-0719-8

**Published:** 2020-01-15

**Authors:** Moritz Jesinghaus, Katja Steiger, Fabian Stögbauer, Bernhard Haller, Andreas Kolk, Ulrich Straßen, Anja Pickhard, Markus Wirth, Miguel Silva, Jan Budczies, Aaron Becker von Rose, Björn Konukiewitz, Peer Kuhn, Konrad Klinghammer, Hendrik Dapper, Stefan Münch, Stephanie E. Combs, Wilko Weichert, Melanie Boxberg

**Affiliations:** 1 Institute of Pathology, Technical University Munich, Munich, Germany; 2 German Cancer Consortium (DKTK), Partner Site Munich, Institute for Translational Cancer Research, Munich, Germany; 3 Institute for Epidemiology and Statistics, Technical University Munich, Munich, Germany; 4 Department of Head and Neck Surgery, Klinikum Rechts der Isar, Munich, Germany; 5 Department of Otolaryngology, Klinikum Rechts der Isar, Munich, Germany; 6 Institute of Pathology, University of Heidelberg, Heidelberg, Germany; 7 III Medizinische Klinik, Klinikum rechts der Isar, Technical University Munich, Munich, Germany; 8 Department for Hematology and Oncology, Charite University Medicine, Berlin, Germany; 9 Department of Radiation Therapy, Klinikum rechts der Isar, Technical University Munich, Munich, Germany; 10 German Cancer Consortium (DKTK), Partner Site Munich, Munich, Germany; 11 Institute of Innovative Radiotherapy (iRT), Helmholtz Center Munich, Munich, Germany

**Keywords:** Head and neck cancer, Tumour biomarkers

## Abstract

**Background:**

Pre-operative treatment planning in head and neck squamous cell carcinoma (HNSCC) is mainly dictated by clinical staging, which has major shortcomings. Histologic grading is irrelevant due to its lack of prognostic impact. Recently, a novel grading termed Cellular Dissociation Grade (CDG) based on Tumour Budding and Cell Nest Size was shown to be highly prognostic for resected HNSCC. We aimed to probe the predictive and prognostic impact of CDG in the pre-operative biopsies of HNSCC.

**Methods:**

We evaluated CDG in *n* = 160 pre-therapeutic biopsies from patients who received standardised treatment following German guidelines, and correlated the results with pre- and post-therapeutic staging data and clinical outcome.

**Results:**

Pre-operative CDG was highly predictive of post-operative tumour stage, including the prediction of occult lymph node metastasis. Uni- and multivariate analysis revealed CDG to be an independent prognosticator of overall, disease-specific and disease-free survival (*p* < 0.001). Hazard ratio for disease-specific survival was 6.1 (11.1) for nG2 (nG3) compared with nG1 tumours.

**Conclusions:**

CDG is a strong outcome predictor in the pre-treatment scenario of HNSCC and identifies patients with nodal-negative disease. CDG is a purely histology-based prognosticator in the pre-therapeutic setting that supplements clinical staging and may aide therapeutic stratification of HNSCC patients.

## Background

Head and neck squamous cell carcinoma (HNSCC) is one of the most common cancers worldwide with ~500.000 new cases annually.^1^ HNSCC arise in different anatomical areas of the head and neck region. Human Papilloma Virus (HPV)— negative cases are particularly located within the oral cavity, hypopharynx and larynx.^2,3^ Overall, the prognosis of HNSCC, especially of HPV-negative cases, is unfavourable with a 5-year survival of patients on average below 50%.^4,5^ To date, tumour stage according to clinical and pathological TNM and UICC classification provides the most important prognostic information for patient outcome.^3–5^ However, pre-operative clinical assessment of tumour size (cT stage) and nodal involvement (cN stage) is discordant from pathological pT- and pN stage in nearly half of cases.^6^ Estimates assume that in early-stage oral squamous cell carcinoma (SCC) without clinically or radiologically apparent nodal metastasis 20–40% of patients contain occult metastasis at the time of diagnosis.^7–9^ As accurate routine biomarkers besides clinical staging for detection of such occult metastasis are still lacking, it is a matter of an ongoing debate which patients could be treated adequately and sufficiently without neck dissection in order to avoid overtreatment and in which patients to perform neck dissection in order to detect clinically non-apparent nodal metastasis and therewith improve prognosis.^10–12^

Currently, histopathologic grading does not play a major role in clinical decision making as current histopathological grading schemes according to the World Health Organization (WHO) possess major shortcomings regarding prognostic patient stratification and reproducibility.^13–17^ In an effort to resolve this issue, our group recently proposed a new histopathological grading scheme termed Cellular Dissociation Grade (CDG) for oral, hypopharyngeal and laryngeal SCC, which is based on the parameters Tumour Budding and Cell Nest Size. These parameters describe the extent of Cellular Dissociation of a given cancer, both from a quantitative (Tumour Budding) and qualitative (Cell Nest Size) angle. CDG has not only been shown to be highly prognostic in other squamous cell carcinomas entities like oesophageal or cervical SCC but was also recently demonstrated to work nicely on pre-therapeutic biopsies of oesophageal SCC.

Biopsies are the mainstay of pre-operative diagnostics in many cancer types, including HNSCC. Pre-therapeutic biopsies are not only necessary to establish a diagnosis but offer the possibility to determine histological subtypes, differentiation and potential further prognostic/predictive information to identify biologically aggressive tumours or to predict treatment response. Therewith, histopathological grading in biopsy specimen guides choice of therapies, including e.g., neoadjuvant systemic treatment versus primary resection or watchful waiting strategies in entities as endometrial cancer and prostate cancer.^18,19^ However, up to date, pre-operative grading in HNSCC is almost meaningless as conventional grading according to the WHO is neither correlated with post-operative tumour stage nor with patient outcome,^13–17^ and is also known to be highly discordant when biopsies and corresponding resection specimen are compared.^20,21^

Based on the above said, major questions remain. Can the high prognostic value of CDG be transferred to small biopsy specimens in HNSCC without losing its prognostic impact concerning patient survival? Can CDG potentially serve as a biomarker for prediction of occult metastasis in cN0 necks in clinically early-stage HNSCC? How is the concordance of CDG when biopsies and corresponding resection specimens are compared? Aiming to answer these questions, we investigated Tumour Budding and Cell Nest Size and CDG derived from these factors in pre-operative biopsies and resection specimen of *n* = 160 patients with HNSCC located in the oral cavity, larynx and hypopharynx. We analysed concordance of CDG between biopsy and resection specimens and correlated CDG with pre-operative clinical staging (cT, cN), post-operative pathological staging (pT, pN) and with patient outcome (overall (OS), disease-specific (DSS) and disease-free survival (DFS)).

## Methods

### Patient cohort

Included in our study were 160 patients with HNSCC of the larynx (supraglottic, glottic, infraglottic primaries), the hypopharynx and the oral cavity from whom pre-operative biopsies and resection specimen were prospectively collected. All patients were treated in curative intent between 2004 and 2015 at Klinikum Rechts der Isar of the Technical University of Munich, Germany. All patients received a complete surgical resection of their primary tumours followed by a neck dissection in combination with radiotherapy (65–70 Gy) or radio-chemotherapy if indicated (pT4 tumours and/or nodal stage pN1 and pN2a/b) according to German guidelines. Exclusion criteria were distant metastasis at the time of diagnosis (cM1 stage), neoadjuvant treatment with radio- and/or chemotherapy and pathological diagnosis upon fresh-frozen tissue sections. Male patients (129/160 (80.6%)) were in the majority compared with female counterparts (31/160 (19.4%)). Mean age at diagnosis was 60.6 years (range: 40.0–87.0 years). Mean follow-up time of patients alive at the endpoint of analysis was 53.5 months, mean survival time of deceased patients 26.9 months. During follow-up, 70/160 (43.8%) of patients died and 65/160 (40.6%) suffered from (local, nodal and/or distant) relapse. Pre-operative clinical staging was performed by clinical examination, ultrasound and/or CT-scan according to the German guidelines.^22^ Clinical and pathological T-stage, N-stage and UICC stage, which were available for 100/160 (62.5%), were documented according to the TNM classification of malignant tumours.^23^ Distribution of clinicopathological parameters is given in Tables 1 and 2. Approval for the study was obtained from the Ethics Review Committee of the Technical University of Munich (296/17s).

**Table 1:** Distribution and survival associations for clinicopathological data, histomorphological parameters and Cellular Dissociation Grade in biopsies and resection specimens.

	Overall	Events (OS)	Mean overall survival (SE)	*p*-value	Events (DSS)	Mean disease-specific survival (SE)	*p*-value	Events (DFS)	Mean disease-free survival (SE)	*p-*value
Overall	160	70	77.3 (5.4)		55	87.5 (5.7)		65	78.7 (5.3)	
*Age*										
Median and below	80	28	83.9 (7.1)	*0.039*	22	91.0 (7.4)	*0.073*	29	82.1 (6.8)	*0.211*
Above median	80	42	66.6 (7.1)		33	79.0 (7.8)		36	73.5 (7.4)	
*Gender*										
Male	129	59	7e.9 (5.7)	*0.301*	45	85.3 (6.1)	*0.663*	52	78.2 (5.9)	*0.870*
Female	31	11	86.1 (12.8)		10	94.2 (12.1)		13	79.5 (12.2)	
*Localisation*										
Infraglottic	11	5	57.3 (10.5)	*0.554*	3	68.8 (10.5)	*0.326*	7	55.1 (10.7)	*0.334*
Glottic	36	13	86.4 (9.2)		9	98.3 (9.1)		11	93.1 (9.1)	
Supraglottic	12	5	89.4 (17.3)		4	96.6 (17.3)		6	88.5 (17.1)	
Hypopharyngeal	41	21	61.6 (7.6)		19	65.2 (7.8)		18	68.1 (7.6)	
Oral	60	26	59.2 (5.3)		20	66.2 (5.3)		23	58.8 (5.9)	
*Histotype*										
Keratinising	126	52	80.4 (6.2)	*0.686*	41	91.2 (6.3)	*0.821*	46	82.6 (6.2)	*0.372*
Non-keratinising	30	16	57.7 (7.8)		12	65.5 (8.6)		16	56.7 (7.6)	
Basaloid	4	2	83.0 (26.6)		2	83.0 (26.6)		3	76.5 (37.2)	
*cT*										
1	16	3	72.4 (5.0)	*0.010*	2	73.3 (5.0)	*0.008*	6	61.2 (5.8)	*0.045*
2	32	7	82.8 (7.7)		4	91.2 (7.2)		6	83.9 (7.8)	
3	23	10	58.4 (9.3)		9	61.3 (9.4)		12	52.7 (8.7)	
4a/b	29	17	55.2 (8.3)		13	63.9 (8.9)		14	63.9 (9.2)	
*cN*										
0	40	10	81.2 (7.1)	*0.066*	7	89.4 (6.5)	*0.023*	12	78.7 (7.0)	*0.029*
1	15	6	65.9 (9.7)		3	79.8 (8.7)		5	68.8 (9.7)	
2	43	19	60.0 (6.6)		16	65.0 (6.6)		19	57.4 (6.9)	
3	2	2	26.0 (5.0)		2	26.0 (5.0)		2	26.0 (5.0)	
*Pre-operative UICC stage*										
1	11	1	80.0 (0.7)	*0.031*	0	Cases censored	*0.021*	4	64.1 (5.7)	*0.040*
2	15	3	86.6 (9.8)		2	91.4 (9.4)		1	98.6 (7.5)	
3	21	7	62.5 (8.0)		5	68.5 (7.8)		8	62.6 (8.1)	
4	53	26	61.3 (6.6)		21	68.5 (6.7)		25	61.7 (7.1)	
*pT*										
1	37	8	101.5 (11.9)	*0.002*	4	124.2 (7.4)	*0.001*	10	98.2 (10.5)	*0.051*
2	52	23	78.8 (8.5)		20	84.1 (8.8)		22	80.6 (8.9)	
3	32	15	62.6 (8.2)		11	73.2 (8.5)		14	67.9 (8.7)	
4a/b	39	24	49.4 (7.2)		20	55.8 (7.7)		19	54.9 (7.7)	
*pN*										
0	73	19	5.8 (6.9)	*<0.001*	11	110.1 (6.2)	*<0.001*	23	86.7 (6.8)	*0.016*
1	26	15	65.0 (11.1)		12	73.9 (12.4)		11	76.3 (13.2)	
2	60	36	57.6 (8.3)		32	62.9 (8.9)		31	68.2 (8.6)	
3	1	0	Cases censored		0	Cases censored		0	Cases censored	
*Post-operative UICC stage*										
1	28	5	90.3 (12.1)	*<0.001*	1	118.6 (4.3)	*<0.001*	6	91.4 (10.3)	*0.010*
2	16	1	123.9 (7.7)		0	Cases censored		5	94.8 (12.0)	
3	36	17	77.0 (10.2)		13	87.3 (10.8)		14	84.6 (10.8)	
4	80	47	58.5 (6.9)		41	64.2 (7.4)		40	66.1 (7.6)	
**Biopsy specimen**										
*Budding/HPF*										
No budding	34	7	99.6 (7.6)	*<0.001*	2	115.5 (5.1)	*<0.001*	4	108.9 (6.3)	*<0.001*
Low budding activity	88	38	73.5 (7.7)		30	82.8 (8.4)		40	69.6 (7.0)	
High budding activity	38	25	50.7 (7.7)		23	53.3 (7.9)		21	53.5 (7.9)	
*Cell nest size*										
Large cell nests	16	4	89.7 (12.8)	*0.004*	1	111.9 (10.3)	*<0.001*	2	102.4 (12.2)	*0.001*
Intermediate cell nests	18	3	97.9 (8.4)		1	108.3 (5.5)		2	106.7 (5.6)	
Small cell nests	55	24	76.7 (9.6)		19	86.8 (10.3)		26	66.7 (8.4)	
Single-cell invasion	71	39	59.2 (7.2)		34	64.8 (7.8)		35	69.2 (8.0)	
*Sum score*										
2	17	4	90.1 (12.8)	*0.001*	1	111.9 (10.3)	*<0.001*	2	102.4 (12.2)	*0.001*
3	17	3	97.6 (8.6)		1	107.9 (5.9)		2	106.4 (5.9)	
5	52	23	76.3 (9.8)		19	84.7 (10.3)		25	66.7 (8.6)	
6	37	15	70.7 (11.3)		11	81.7 (12,8)		16	73.6 (11.6)	
7	37	25	48.8 (7.7)		23	51.4 (8.0)		20	55.0 (8.1)	
*Cellular dissociation grade*										
nG1	34	7	99.6 (7.6)	*<0.001*	2	115.5 (5.4)	*<0.001*	4	108.9 (6.3)	*<0.001*
nG2	89	38	74.0 (7.7)		30	83.3 (8.4)		41	69.0 (6.9)	
nG3	37	25	48.8 (7.7)		23	51.4 (.0)		20	55.0 (8.1)	
**Resection specimen**										
*Budding/10 HPF*										
No budding	31	6	101.2 (7.8)	*<0.001*	1	119.5 (3.5)	*<0.001*	3	112.5 (5.4)	*<0.001*
Low budding activity	85	35	76.6 (8.2)		27	87.0 (9.0)		36	72.3 (7.6)	
High budding activity	44	29	49.1 (7.0)		27	51.2 (7.2)		26	51.3 (6.9)	
*Cell nest size*										
Large cell nests	6	1	67.0 (7.8)	*0.001*	0	Cases censored	*<0.001*	1	72.0 (2.8)	*<0.001*
Intermediate cell nests	26	5	102.9 (8.0)		1	118.9 (4.0)		2	115.6 (4.8)	
Small cell nests	45	16	82.2 (11.3)		13	93.4 (10.9)		19	73.9 (10.3)	
Single-cell invasion	83	48	54.2 (5.4)		41	59.4 (5.9)		43	56.2 (5.2)	
*Sum score*										
2	6	1	67.0 (8.0)	*0.001*	0	Cases censored	*<0.001*	1	72.0 (2.8)	*<0.001*
3	25	5	101.3 (8.6)		1	118.6 (4.3)		2	115.2 (5.1)	
5	45	16	84.1 (11.0)		13	94.5 (10.8)		18	78.0 (10.4)	
6	41	19	52.0 (4.4)		14	57.4 (4.5)		19	51.7 (4.2)	
7	43	29	48.1 (7.0)		27	50.3 (7.2)		25	51.9 (7.1)	
*Cellular dissociation grade*										
nG1	31	6	101.2 (7.8)	*<0.001*	1	119.5 (3.5)	*<0.001*	3	112.5 (5.4)	*<0.001*
nG2	86	35	77.0 (8.3)		27	87.4 (9.0)		37	71.8 (7.5)	
nG3	43	29	48.1 (7.0)		27	50.3 (7.2)		25	51.9 (7.1)	

The values given in italics are the *p*-values obtained in statistical analysis for the respective correlation

**Table 2:** Correlation of histomorphological parameters and cellular dissociation grade with clinicopathological data.

	Cell Nest Size					Budding activity				Grading			
	Large	Intermediate	Small	Single-cell invasion	*p*-value	Absent	Low	High	*p*-value	nG1	nG2	nG3	*p*-value
*Age*													
Median and below	6	11	28	35	*0.589*	17	43	20	*0.927*	17	44	19	*0.981*
Above median	10	7	27	36		17	45	18		17	45	18	
*Gender*													
Male	13	18	41	57	*0.131*	31	66	32	*0.104*	31	67	31	*0.117*
Female	3	0	14	14		3	22	6		3	22	6	
*cT stage*													
1	3	1	11	1	*0.017*	4	11	1	*0.059*	4	11	1	*0.100*
2	3	5	12	12		8	21	3		8	21	3	
3	3	2	6	12		5	9	9		5	10	8	
4a/b	1	3	6	19		4	15	10		4	15	10	
*cN stage*													
0	4	6	18	12	*0.010*	10	23	7	*0.447*	10	23	7	*0.404*
1	1	4	6	4		5	7	3		5	8	2	
2	5	1	9	28		6	24	13		6	24	13	
3	0	0	2	0		0	2	0		0	2	0	
*Pre-therapeutic UICC stage*													
I	2	1	8	0	*0.073*	3	8	0	*0.142*	3	8	0	*0.231*
II	1	3	6	5		4	10	1		4	10	1	
II	2	3	7	9		5	8	8		5	9	7	
Iva/b	5	4	14	30		9	30	14		9	30	14	
*pT stage*													
1	7	4	19	7	*0.001*	11	23	3	*0.002*	11	23	3	*0.002*
2	2	8	18	24		10	32	10		10	33	9	
3	6	4	3	19		10	9	13		10	9	13	
4a/b	1	2	15	21		3	24	12		3	24	12	
*pN stage*													
0	12	12	31	18	*<0.001*	24	39	10	*0.001*	24	39	10	*0.001*
1	2	2	9	13		4	16	6		4	17	5	
2	2	4	14	40		6	32	22		6	32	22	
3	0	0	1	0		0	1	0		0	1	0	
*Post-therapeutic UICC stage*													
I	7	3	15	3	*0.001*	10	16	2	*0.003*	10	16	2	*0.005*
II	1	4	5	6		5	11	0		5	11	0	
II	5	5	9	17		10	15	11		10	16	10	
Iva/b	3	6	26	45		9	46	25		9	46	25	
*Recurrence*													
No recurrence	14	16	28	36	*0.248*	30	47	17	*0.321*	30	47	17	*0.394*
Local recurrence	1	2	11	8		3	14	5		3	14	5	
Nodale recurrence	0	0	5	1		0	6	0		0	6	0	
Distant recurrence	1	0	4	15		1	11	8		1	11	8	
Combined recurrence	0	0	6	9		0	8	7		0	9	6	
Recurrence; localisation unclear	0	0	1	2		0	2	1		0	2	1	

The values given in italics are the *p*-values obtained in statistical analysis for the respective correlations

### Histopathological evaluation

Biopsy and resection specimen from all 160 patients were available. Full block haematoxylin and eosin (H&E)-stained slides of biopsy specimen were evaluated simultaneously by experienced pathologists (MB., MJ.) blinded to clinicopathological data and follow-up using an Olympus BX43 microscope with a field diameter of 0.55 mm (0.24 mm²). Resection specimen had previously been evaluated in the context of two recently published studies.^17,24^ In case of discordance between biopsy and resection specimen, both biopsy and resection specimen were comparatively re-evaluated. HNSCC were categorised according to the current WHO classification criteria of tumours of head and neck into non-keratinising, keratinising and basaloid.^3^

Tumour Budding and Cell Nest Size and subsequent CDG were evaluated as described before in detail.^17,24,25^ In short, Tumour Budding was defined as “branching” of small tumour nests harbouring <5 tumour cells into the stroma/parenchyma,^17,26,27^ and was assessed in one high-power-field (HPF) in biopsies and in ten (continuous) HPFs in resection specimen in areas showing maximal budding activity. The slight modification of the evaluated tumour area (one HPF in biopsies versus ten HPFs in resection specimen) was necessary due to the limited tissue availability in biopsy samples. Low budding activity was defined as 1–4 buds in one HPF (biopsies) and as 1–14 budding nests in ten HPFs (resections), high budding activity was defined as ≥5 in one HPF (biopsies) and as ≥15 budding nests in ten HPFs (resections). Overall Cell Nest Size, a parameter additionally measuring the dissociative growth capability of an individual tumour from a qualitative point of view, was evaluated in tumour area containing smallest tumour cell nests. Tumour cell nests, defined as clustered tumour cells surrounded by tumour stroma, were classified according the size of the smallest invasive cell nest as follows: > 15 tumour cells = large nests, 5–15 tumour cells = intermediate nests, 2–4 tumour cells = small nests, discohesive tumour cells without nested architecture = single-cell invasion. The criteria for budding activity and cell nest size evaluation applied were similar to already established algorithms for the evaluation of CDG in biopsies and resection specimen. According to our previously proposed grading system,^17,24,25^ scores for budding activity (1 = no budding activity; 2 = low budding activity; 3 = high budding activity) and cell nest size (1 = large cell nests; 2 = intermediate cell nests; 3 = small cell nests; 4 = single-cell invasion) were assigned in order to obtain the CDG. After summarising both scores into a sum score from 2 to 7, HNSCC were graded into well cohesive (CDG nG1; sum scores 2, 3), moderately cohesive (CDG nG2; sum scores 4–6) and poorly cohesive (CDG nG3; sum score 7) according to the CDG.

### Statistics

Associations of grouped morphological characteristics with clinicopathological parameters were calculated with Χ^2^ test as well as Χ^2^ test for trends and Fisher’s exact test. Cohen’s kappa was used to assess concordance between clinical and pathological staging parameters as well as to assess concordance between histopathological parameters in biopsies and resection specimen. Survival probabilities were plotted with the Kaplan–Meier method, a log-rank test was used to probe for the significance of differences in survival. Multivariate survival analysis was performed with the Cox proportional hazard model. *P*-values ≤ 0.05 were considered significant. All statistical tests were performed two-sided, and a significance level of 5% was used.

## Results

### Tumour Budding and Cell Nest Size in pre-therapeutic biopsy specimens

**Fig. 1:**
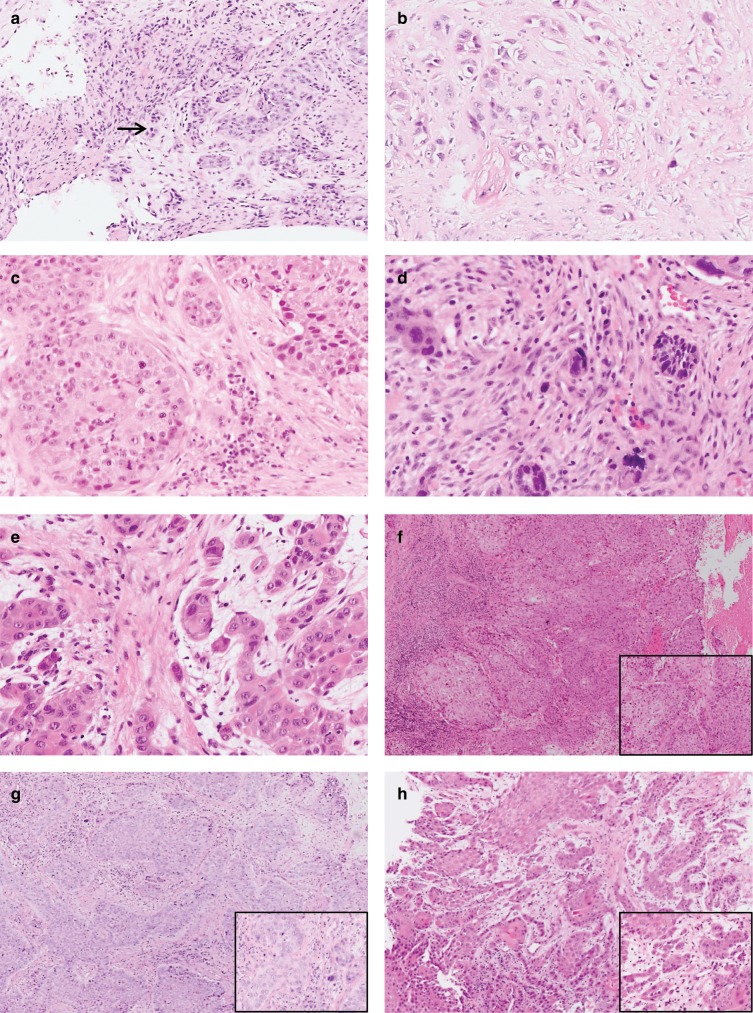
Representative H&E stained biopsy specimen of head and neck squamous cell carcinomas (HNSCC). **a** depicts a HNSCC with low Tumour Budding as indicated by presence of <5 cell nests containing <5 tumour cells within one HPF (→ highlighting the two tumour buds), **b** depicts a HNSCC with high Tumour Budding as indicated by presence of ≥5 cell nests containing <5 tumour cells within one HPF (→ highlighting some of the numerous tumour buds of this carcinoma). **c**–**e** depict different Cell Nest Sizes with **C** showing large (*) and intermediate (→) cell nest sizes, while **d** + **e** show carcinomas with small cell nests (*) and single-cell invasion (→). **f**–**h** Overview over representative HNSCC biopsies with **f** showing Cellular Dissociation Grade nG1 carcinoma (no Tumour Budding, large Cell Nest Size), **g** showing a Cellular Dissociation Grade nG2 carcinoma (low Tumour Budding, small Cell Nest Size) and **h** showing a Cellular Dissociation Grade nG3 carcinoma (high Tumour Budding, single-cell invasion). Inserts showing the HPF[×40] of the respective cases with the highest Tumour Budding activity and the smallest Cell Nest Size within the biopsy specimen.

The majority of HNSCC were keratinising tumours (126/160, 78.8%), whereas non-keratinising cancers (30/160, 18.8%) and basaloid cancers (4/160; 2.4%) were less frequent. No tumour budding activity (per HPF) was detected in 34/160 (21.3%), while low budding activity was observed in 88/160 (55.0%) of cases and high budding activity in 38/160 (23.7%) of cases. Analysis of the Cell Nest Size revealed large cell nests in 16/160 cases (10.0%), intermediate nests in 18/160 cases (11.3%) and small cell nests in 55/160 cases (34.3%). In total, 71/160 cases (44.4%) showed infiltrative singular tumour cells, and were therefore classified as single-cell invasion. Addition of scores obtained for budding activity (scores 1–3) and cell nest size (scores 1–4) resulted in a sum scores 2–7 for each case, which was assigned to the final CDG (nG1, nG2, nG3) as described in the Methods section. While 34/160 (21.3%) HNSCC were well differentiated (nG1), 89/160 (55.6%) were moderately differentiated (nG2) and 37/160 (23.1%) poorly differentiated (nG3; Table 1; Fig. 1) according to the CDG.

### Concordance of the Cellular Dissociation Grade between pre-therapeutic biopsies and resection specimens

The resection specimen of this cohort have previously been evaluated in two studies applying a similar grading algorithm.^17,24^ Comparison of Tumour Budding in the corresponding biopsies and resection specimen revealed a high concordance with similar scores obtained in 147/160 (91.9%) cases. The corresponding Kappa value was κ = 0.87. Concerning Cell Nest Size, concordance rate was considerably lower with 102/160 (63.8%) HNSCC showing the same cell nest size in biopsy and resection specimen (Kappa value κ = 0.44). Analysis of concordance rate between assigned grades resulted in 147/160 (93.1%) HNSCC with identical histopathological grade (Kappa value κ = 0.89). Of the 13 cases with discordant grading, which was confirmed upon re-evaluation of biopsy and resection specimen, 12 showed an upgrading whereas only one case was downgraded from nG2 to nG1 (Supplementary Table 1). Upgrading was observed in the setting of very small tumour-containing biopsy specimen (<0.3 cm). Downgrading was detected in a large biopsy specimen of a pT1 carcinoma, which contained a majority of the whole tumour leaving only small remnants of the invasive tumour within the resection specimen.

### Correlation of Tumour Budding, Cell Nest Size and Cellular Dissociation Grade in biopsies with pre-operative staging

Cell nest size was significantly correlated with cT (*p* = 0.017) and cN (*p* = 0.010) stage with single-cell invasion being more frequently detected in HNSCC patients with higher clinical stages. No further significant correlation of either budding activity or overall grading with clinical staging parameters were observed (Table 2).

### Correlation of Tumour Budding, Cell Nest Size and Cellular Dissociation Grade in biopsies with post-operative staging

**Fig. 2:**
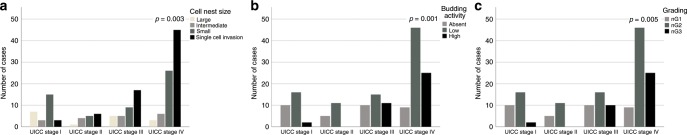
Associations of UICC stage with histomorphological parameters and Cellular Dissociation Grade. Association between UICC tumour stage with Cell Nest Size (**a**), Tumour Budding (**b**) and Cellular Dissociation Grading **(c**).

Cell Nest Size and Tumour Budding as well CDG were significantly correlated with post-operative pathological staging parameters pT stage, pN stage and UICC stage. Presence of small cell nest sizes and single-cell invasion as well as presence of budding activity were more frequently detected in cases with higher pT, pN and UICC stage (*p* < 0.005). This was mirrored by correlation of moderate (nG2) and poor (nG3) differentiation with higher post-operative stages (*p* < 0.01; Table 2, Fig. 2).

### Concordance of pre-therapeutic and post-therapeutic staging parameters

Clinical cT and post-operative pT stage was discordant in 36/100 (36.0%) HNSCC (Kappa value κ = 0.52). In total, 14 cases received an upstaging and 22 a downstaging after pathological evaluation of the resection specimen. Respective values for nodal stage and UICC stage were as follows: discordance rate cN vs. pN 32/100 (32.0%; κ = 0.49); pre-operative UICC vs. post-operative UICC 33/100 (33.0%; κ = 0.49). Upstaging: nodal stage 12 cases; UICC stage 14 cases. Downstaging: nodal stage 20 cases; UICC stage 19 cases (Supplementary Table 2).

### Subgroup analysis of HNSCC with clinically nodal-negative (cN0) necks: correlation of Cellular Dissociation Grade in biopsies with post-operative staging parameters

HNSCC patients with nodal-negative cervical lymph nodes upon clinical staging procedures (cN0 necks; *n* = 40) showed occult metastases detected by pathological evaluation of neck dissection specimen in 8/40 (20.0%) cases. Histopathological grading of all of these cases was nG2/3, whereas in none of the nG1 cases presence of lymph node metastases was observed. This result indicated a positive predictive value PPV = 100% for nG1 grading to predict nodal negativity upon pathological work-up in cN0 necks. Association of grading with post-operative nodal stage is summarised in Supplementary Table 3.

### Correlation of Tumour Budding, Cell Nest Size and Cellular Dissociation Grade in biopsies with patient survival

Tumour budding activity was strongly associated with decreased OS, DSS and DFS (*p* < 0.001, respectively) with HNSCC with high budding activity revealing the shortest mean OS. In analogy, Cell Nest Size was strongly correlated with OS, DSS and DFS (*p* < 0.01, respectively). Cases with single-cell invasion and small cell nests suffered from earlier deaths and tumour recurrences compared with their counterparts (Table 1; Supplementary Fig. 1). Not only both morphologic patterns but also the obtained sum score was significantly correlated with survival parameters of patients (*p* < 0.005; Table 1).

**Fig. 3:**
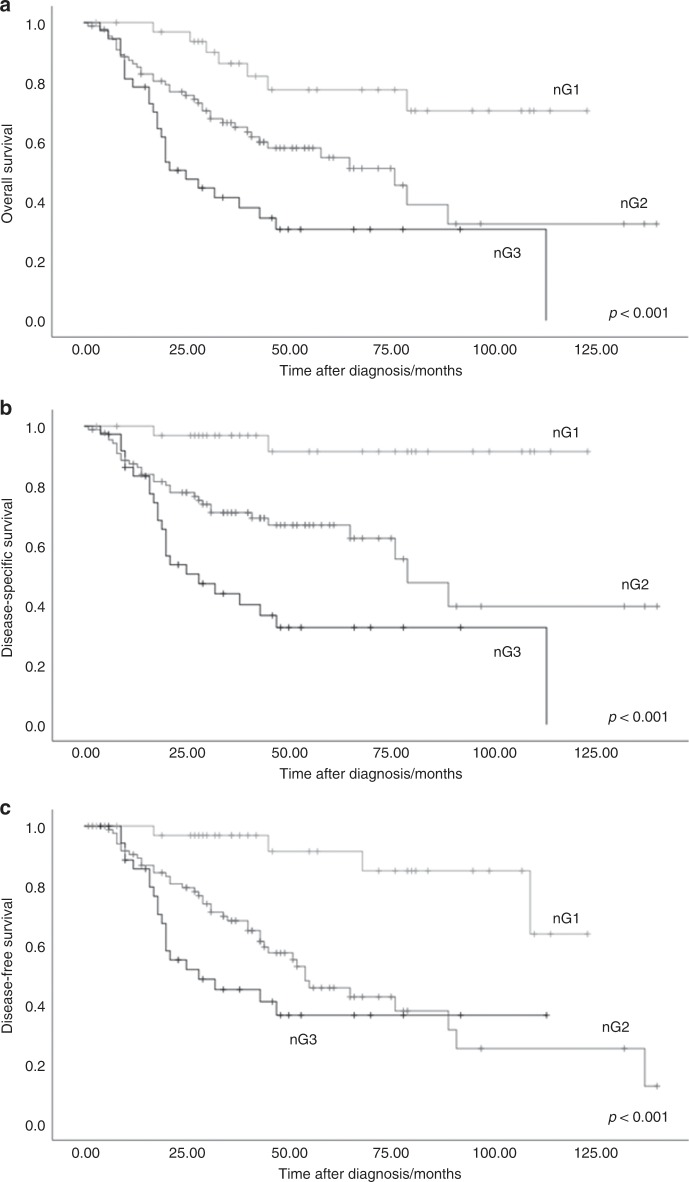
Association of Cellular Dissociation Grade with survival. **a** Overall survival, **b** disease-specific survival, **c** disease-specific survival.

Consequently, the CDG on pre-therapeutic biopsies which is based on the sum scores also showed a highly significant impact on OS, DSS and DFS (*p* < 0.001, respectively) (Table 1, Fig. 3). While mean DSS of nG1 tumours was 119.5 months, it dropped to 87.4 months for nG2 HNSCC and to 50.3 months for nG3 HNSCC (Table 1).

In order to prove that the prognostic impact of the novel grading system derived from biopsy specimens is independent from clinicopathological data and tumour staging, we performed a multivariate analysis by applying a cox regression model incorporating pre-operative, respectively, post-operative staging parameters (cUICC/pUICC), gender and age and the CDG. Multivariate analysis confirmed the independent prognostic value of the novel grading concerning patient survival (OS, DSS, DFS; Table 3 for cox regression with pUICC; Supplementary Table 4 for cox regression with cUICC). Hazard ratio for DSS was 6.1 (95% confidence interval 1.4–26.4) for nG2 and 11.1 (95% confidence interval 2.6–48.0) for nG3 HNSCC compared to nG1 cases (*p* = 0.002; multivariate analysis including pUICC).

**Table 3:** Multivariate survival analysis including Cellular Dissociation Grade, age, gender and post-operative UICC stage.

	Hazard ratio	Confidence interval		*p*-value
	HR	Low	High	
**Overall survival**				
*Gender*				
Male	1.000			*0.160*
Female	0.625	0.325	1.204	
*Age*				
Median and below	1.000			*0.022*
Above median	1.764	1.087	2.880	
*UICC stage*				
I	1.000			*0.008*
II	0.298	0.034	2.574	
III	2.043	0.740	5.644	
Iva/b	3.194	1.250	8.164	
*Cellular Dissociation Grade*				
nG1	1.000			*0.004*
nG2	2.500	1.078	5.797	
nG3	4.222	1.771	10.065	
**Disease-specific survival**				
*Gender*				
Male	1.000			*0.417*
Female	0.750	0.375	1.502	
*Age*				
Median and below	1.000			*0.054*
Above median	1.717	0.991	2.973	
*UICC stage*				
I	1.000			*0.046*
II	<0.001	<0.001	>100.0	
III	7.462	0.962	57.875	
Iva/b	12.382	1.690	90.713	
*Cellular Dissociation Grade*				
nG1	1.000			*0.002*
nG2	6.131	1.426	26.361	
nG3	11.095	2.565	47.999	
**Disease-free survival**				
*Gender*				
Male	1.000			*0.486*
Female	0.802	0.431	1.492	
*Age*				
Median and below	1.000			*0.130*
Above median	1.478	0.892	2.451	
*UICC stage*				
I	1.000			*0.136*
II	1.123	0.336	3.751	
III	1.452	0.543	3.883	
Iva/b	2.245	0.939	5.364	
*Cellular Dissociation Grade*				
nG1	1.000			*0.002*
nG2	5.002	1.746	14.336	
nG3	7.393	2.456	22.260	

The values in italics represent the *p*-values for the respective statistical analysis

## Discussion

Pre-operative biopsy is the gold standard for cancer diagnosis in many cancers, including HNSCC. Ideally, histomorphologic diagnosis and subsequent histopathologic grading upon biopsy enables a prognostic patient stratification by distinction of cancers with comparably benign clinical course from those with aggressive tumour biology. In many entities including prostate and endometrial cancer, pre-operative biopsies therewith enable guidance of therapeutic approaches and clinical decision making, including the choice for neoadjuvant treatments or the selection of watch and wait strategies.^18,19^ In contrast, studies in HNSCC revealed, that conventional histopathologic grading as defined by WHO criteria in incisional biopsies lacks impact on clinical outcome,^20,21^ which is in line with studies that reported WHO grading to be of only limited prognostic relevance in resection specimen.^13–17^ Another important pre-exquisite—if therapy decisions should be based on biopsy readouts—is to ensure the representativity of the data which can be obtained from biopsies. Thus, a high concordance of grading in biopsies and corresponding resection specimen is desirable, as discordances can potentially lead to erroneous decisions and treatment failure. Current WHO grading in HNSCC biopsies not only has limited prognostic impact but also fails to meet this criterion as Dik et al. recently found major discordance of grading between biopsy and corresponding resection specimen for this scheme.^20,21^

We recently proposed a novel grading algorithm in HNSCC with high prognostic impact^17,24^ and high inter- and intra-observer concordance^25^ termed Cellular Dissociation Grade, which has also been shown to work nicely in other SCC entities including in pre-therapeutic biopsies of oesophageal SCC. The CDG is based on the cellular dissociation parameters Tumour Budding and Cell Nest Size, which are both histomorphologic readouts measuring the capability of dissociative growth of an individual carcinoma from either a quantitative (Tumour Budding) or qualitative (Cell Nest Size) point of view. Considering the above discussed potential major prognostic and predictive implications of the data derived early in the diagnostic process from biopsy specimens, we aimed to probe if the prognostic value of the CDG is retained when assessed in pre-therapeutic biopsies of patients suffering from HNSCC. In addition, we analysed the value of the pre-operative CDG for the prediction of post-operative tumour stage, including the presence of occult lymph node metastasis in clinically and radiologically negative cN0 necks.

We found, that Tumour Budding and Cell Nest Size are of major prognostic relevance for patient outcome with presence of high budding activity and single-cell invasion indicating the most aggressive disease course with early relapses and poor survival. Summarising these two parameters and applying our CDG (nG1, nG1, nG3) discriminated three distinct prognostic groups. The prognostic value of the grading system was independent of potential additional influencing factors, such as age, gender and stage and revealed hazard ratios for OS (DSS; DFS) for nG2/nG3 HNSCC of 2.5 (6.1; 5.0)/4.2 (11.1; 7.4). Again, previous studies revealed that the current conventional WHO-based grading of HNSCC does not show prognostic impact when applied in biopsy specimens.^20,21^ In addition, the Kappa value κ = 0.89 for the corresponding biopsy and resection specimen grading with 94% concordantly graded HNSCC proves the representativity of the biopsy grading results for the overall tumour configuration. Of 160 HNSCC, an upgrading from biopsy to resection specimen was only observed in ten cases. In all of these cases, the biopsies only harboured very superficially captured carcinoma. In only one case, downgrading was evident. Review of both specimens revealed that this case was a T1 HNSCC, in which a large incisional biopsy comprised the major part of the carcinoma leaving only residual invasive cancer within the resection specimen. Regarding the concordance of the two parameters budding and cell nest size in biopsy and the corresponding resection specimen, concordance rate of the morphologic pattern tumour budding was strikingly higher (κ = 0.87) compared with the pattern cell nest size (κ =  0.44). While tumour budding provides an overview of the dissociative growth capability, cell nest size represents the highest dissociative capability within the area of highest aggressiveness. This might be the reason why tumour heterogeneity plays a more prominent role in the evaluation of the cell nest size: biopsies by nature only capture a smaller part of carcinomas compared to resection specimen, potentially leading to a bias induced by tumour heterogeneity when evaluating cell nest size as the area of highest dissociative capability might not be included in the biopsy specimen. However, this bias particularly comes to effect in cases with scores 2/3 for cell nest size, and therewith obviously does not lead to a major effect on the sum score and the final grade, which is demonstrated by the high kappa value for the overall grading. Dik et al. analysed concordance of WHO-based grading of HNSCC between biopsy and resection specimens and found >50% discordantly graded cases in their studies.^20,21^ Concordance rates of histopathological grading in other entities as prostate cancer were higher with 50–70% concordant cases.^28,29^ The concordance rate of our grading is surprisingly high, which might be due to large and representative incisional biopsies performed at our institution.

Besides our grading approach, several alternative HNSCC grading systems have been proposed, including the “malignancy grading of invasive margins” (MG),^30^ the “histological risk model” (HR)^15^ and the “tumour budding and depth of invasion” (BD)^31^ grading system. In contrast to our CDG, which has been successfully applied in oral SCC,^17,25^ in laryngeal and in hypopharyngeal SCC,^24^ and which has been transvalidated in oesophageal,^26^ cervical^32^ and pulmonary SCC,^27^ all of these approaches yielded varying results with respect to prognostic value and interobserver variability.^15,16,30,31,33–37^ Only the prognostic impact of the BD grading system, which evaluates BA together with tumour invasion depth, was confirmed in recent studies.^16,31^ However, per definition tumour invasion depth is a parameter that rather describes tumour stage than tumour grade as defined by the current TNM manual.^23,38,39^ Furthermore, this parameter is not applicable in biopsies, limiting its value for routine biopsy diagnostics.^38,40^

Incorporation of markers of cellular dissociative capability such as Tumour Budding and Cell Nest Size into a grading system seems reasonable as presence of Tumour Budding has been shown to be associated with an unfavourable disease course not only in HNSCC (reviewed in refs. ^41,42^) but also in other solid tumours, such as colorectal^43,44^ and pulmonary carcinomas.^27^ Confirming our results, Tumour Budding has been shown to be of prognostic value not only in resection but as well in biopsy specimen of these tumour entities (reviewed in ref. ^45^). Focusing on the biologic background of Tumour budding, recent research has shown, that this histologic pattern might be the morphologic representative of epithelial-mesenchymal transition (reviewed in ref. ^46^). It has been identified as a sign of invasion and metastasis and has therefore been proposed as a novel hallmark of cancer by Makitie et al. (reviewed in ref. ^47^).

Both, Tumour Budding and Cell Nest Size as well as the composite parameter CDG when assessed in pre-operative biopsies were strongly correlated with post-operative pT and pN stage. Therefore, post-operative positive nodes were significantly more frequent in cases with a moderate or poor tumour differentiation (nG2/nG3) according to the CDG compared with well-differentiated carcinomas. In contrast, only Cell Nest Size but not Tumour Budding or CDG was correlated with pre-operative staging parameters (cT/cN). This might be due to the high discordance rate of pre-operative clinical cT and cN stage compared with post-operative pT and pN in 36%, respectively, 32% cases—a finding which confirms previous studies in HNSCC studies showing comparably low concordance rates between clinical and pathological staging.^6,48,49^ Furthermore, in our study, the subgroup of clinically early HNSCC in UICC stages I and II 20% of cN0 necks revealed occult nodal metastasis only detected by pathological examination. Considering the ongoing discussion if clinically nodal-negative necks should be treated by elective neck dissection or by watchful waiting strategies, these results support the notion and confirm previous studies that clinical staging in its current form is not a valuable predictive tool for treatment planning in these patients,^10,11,50,51^ as it lacks the necessary sensitivity for detection of metastatic disease. Searching for a better biomarker for the presence of occult nodal metastasis in these patients, Sakata et al. proposed Tumour Budding as a novel predictor of occult lymph node metastasis in oral SCC^12^ in a recent study. In our study—confirming this result—none of clinically cN0-staged patients with nG1 tumour differentiation according to the CDG revealed an occult nodal metastasis upon pathological work-up, whereas occult metastasis was detected in 36% of nG2/nG3 early-stage patients. Our study therewith underlines the potential major importance of Tumour Budding as a predictive biomarker for lymph node metastasis in cN0 necks. The predictive value could potentially be further increased by combining CDG assessment on biopsies with modern imaging modalities such as PET CT.^52^

Our study clearly has some limitations as the included patient cohort contained a majority of advanced HNSCC. Furthermore, the study cohort included cases from different areas of the head and neck region (oral cavity, larynx, hypopharynx). The study design was based on a prospective-retrospective concept with prospective sample collection from the day of the biopsy/resection followed by retrospective histopathological evaluation. Therefore, additional especially prospective study concepts and multi-centre studies are necessary to validate the prognostic impact of CDG in the pre-operative setting of HNSCC.

Taken together, our study revealed that CDG based on Tumour Budding and Cell Nest Size can successfully be applied in pre-therapeutic biopsies of HNSCC. The high prognostic impact of CDG assessed in biopsy specimen, separating three distinct prognostic groups, was independent of other prognostic parameters. Pre-operative grading was furthermore predictive for the presence of occult nodal metastasis, which had not been detected by routine clinical staging procedures in clinically nodal-negative necks. Considering the prognostic impact in the post-therapeutic setting and the high inter- and intra-observer reliability of the CDG which we demonstrated in previous studies,^17,25^ we believe, that grading of HNSCC by CDG shows a promising potential for applicability in daily routine pathological and clinical practice. Taking together our previous studies,^17,24,25^ the major prognostic impact of the CDG was shown in the pre- and post-operative setting in the same homogeneous patient cohort as was the high inter- and intra-observer reproducibility of the grading system. Using the same patient cohort may prove the validity and significance of the results, as it allows to exclude a coincidental effect which might be observed when using different study cohorts.

In conclusion, we believe that our findings provide a rationale for the integration of CDG assessment in routine diagnostics of HNSCC biopsies, since CDG has the potential to play a substantial role in treatment planning and prognostic patient stratification in HNSCC in the pre-therapeutic setting.

## Supplementary information

Supplementary Material

## Data Availability

All data relevant for this study are given with the main paper, including figures, tables and the supplementary files. The tissue investigated for this study is archived in the Institute of Pathology of the Technical University of Munich.
